# The aging biological clock in *Neurospora crassa*

**DOI:** 10.1002/ece3.1202

**Published:** 2014-08-22

**Authors:** Mary E Case, James Griffith, Wubei Dong, Ira L Tigner, Kimberly Gaines, James C Jiang, S Michal Jazwinski, Jonathan Arnold, for the Georgia Centenarian Study

**Affiliations:** 1 Department of Genetics, University of GeorgiaAthens, Georgia, 30602; 2 College of Agricultural and Environmental Sciences, University of GeorgiaAthens, Georgia, 30602; 3 Tulane Center for Aging and Department of Medicine, Tulane University Health Sciences CenterNew Orleans, Louisiana, 70112

**Keywords:** Aging, *bd*, biological clock, *lag-1*, *Neurospora crassa*, *ras-1*

## Abstract

The biological clock affects aging through *ras-1* (*bd*) and *lag-1*, and these two longevity genes together affect a clock phenotype and the clock oscillator in *Neurospora crassa*. Using an automated cell-counting technique for measuring conidial longevity, we show that the clock-associated genes *lag-1* and *ras-1* (*bd*) are true chronological longevity genes. For example, wild type (WT) has an estimated median life span of 24 days, while the double mutant *lag-1, ras-1* (*bd*) has an estimated median life span of 120 days for macroconidia. We establish the biochemical function of *lag-1* by complementing *LAG1* and *LAC1* in *Saccharomyces cerevisiae* with *lag-1* in *N. crassa*. Longevity genes can affect the clock as well in that, the double mutant *lag-1*, *ras-1* (*bd*) can stop the circadian rhythm in asexual reproduction (*i.e*., banding in race tubes) and lengthen the period of the *frequency* oscillator to 41 h. In contrast to the *ras-1* (*bd), lag-1* effects on chronological longevity, we find that this double mutant undergoes replicative senescence (*i.e*., the loss of replication function with time), unlike WT or the single mutants, *lag-1* and *ras-1* (*bd*). These results support the hypothesis that sphingolipid metabolism links aging and the biological clock through a common stress response

## Introduction

In a number of model systems, the network of genes under the control of the biological clock has been found to be quite large. In one model fungal system, *N. crassa*, up to 25% percent of the genes in the genome appeared to be circadian in expression (Dong et al. 2008). With such far-reaching effects by the clock on the organism's transcriptome, it is natural to ask whether or not there is a link between the clock and aging (Chen et al. 2008). One of the earliest studies of the clock's effect on longevity was in *Drosophila melanogaster* (Pittendrigh and Minis 1972*)*. Flies reared on their natural 24-hour day lived longer than flies reared on other light/dark (L/D) cycles. In the nematode, *Caenorhabditis elegans* clock genes were shown to have a direct impact on life span (Lakowski and Hekimi 1996). More recently, in mice, the sirtuin protein SIRT1 encoded by a major longevity gene (Kim et al. 2012) promoted the deacetylation and degradation of PER2 in mouse, and this protein SIRT1 was required for high-magnitude circadian expression of the clock mechanism genes, *BMAL1, PER2*, and *CRY1* (Asher et al. 2008*)*.

In this work, we examine the connection between the clock and aging in *N. crassa* and its relatives, which have given us many insights into the molecular mechanisms of the clock (Dunlap 1999) (Brunner and Kaldi 2008) and aging (Munkres and Furtek 1984c) (Munkres and Furtek 1984b) (Munkres and Furtek 1984a) (Griffiths 1992).

There are a variety of reasons to expect that aging and circadian rhythms should be functionally connected. One view of circadian rhythms is that they can provide a response to periodically recurring stresses (Bennett et al. 2013). On the other hand, one of the major theories of aging is that the aging process is a response to stress as well (Hagberg 2007). In either case, the mobilization of an organism's metabolic reserves should be involved and hence there should be a connection of lipid metabolism to both circadian rhythms and aging. We hypothesize that the underlying clock mechanism should have an impact on aging through its control of lipid metabolism (Lakin-Thomas and Brody 2000); likewise, genes that are involved in controlling metabolic reserves, such as those in lipid metabolism, should also have an impact on the ability of the organism to respond to periodic stresses through circadian rhythms. The intimate connection between circadian rhythms and aging has long been appreciated through the circadian control of diapause, a trait that has strong connections to life span and responses to environmental stress (Meuti and Denlinger 2013).

**Figure 1:**
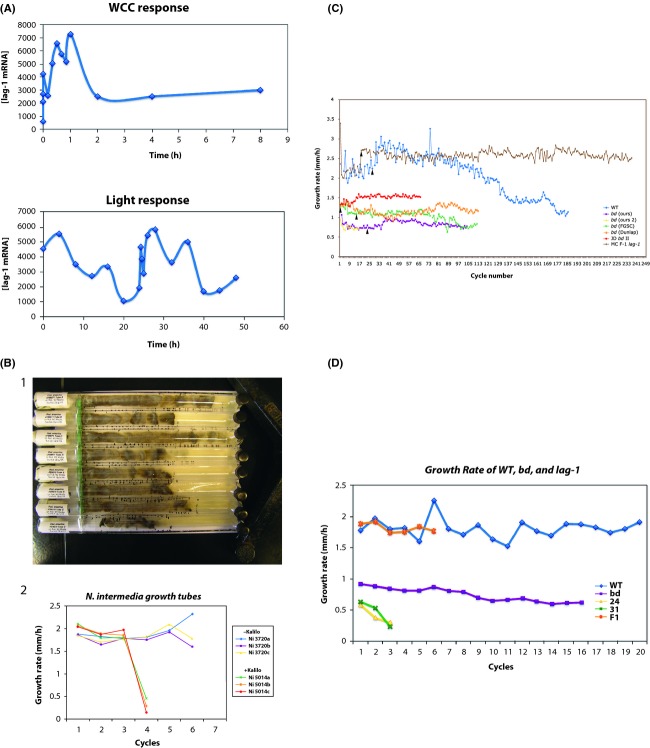
(A) WCC response and light response to *lag-1*^*+*^ mRNA levels in cycle 3 and cycle 2 microarray experiments (Dong et al. 2008). Both responses are significant at the *α* = 0.20 level. The t-tests are described in detail in the legends of figs 10 and 13 (Dong et al. 2008). While the *lag-1*^*+*^ did not appear to have a canonical WCC-binding site, it did survive a periodicity test after a Benjamini–Hochberg multiple test correction with nominal alpha of 0.05 in cycle 1 (in the dark) (See legend to fig. 6 in Dong et al. 2008). Raw data for this metanalysis are deposited (Zhang and Townsend 2010). (B) Some strains of *P. anserina* and *N. intermedia* senesce in serial transfer experiments. 1. Strains +F89071 and –F89071 of *P. anserina* (with + and – indicating mating type) senesce within one cycle of serial transfer, although replicates shown differ in growth rate. 2. Strains 3720 and 5014 (Kalilo) differ in the absence versus presence of an extrachromosomal plasmid that inserts into the mitochondrial DNA causing senescence. Three replicates of the Kalilo strain senesce after 4 cycles of serial transfer. (C) *N. crassa* cultures can be maintained by serial transfer between race tubes for over 60 cycles from the plugs of tubes without light on QA (0.001 mol/L). Several *bd* mutants with slightly different genetic backgrounds (ours, FGSC, Dunlap (328-4)) were used. There is one replicate of 328-4 and our *bd* (ours 2 and JD *bd* II). WT and a *lag-1* (NCU00008 F1-1) strain were similarly maintained. The switch from conidial washes to plugs as inoculum for the beginning of each cycle began at cycle: (WT) 31; (F1) 19; (*bd* ours) 23; (*bd* FGSC) 17; (*bd* Dunlap) 1. Black triangles indicate where serial transfer with plugs begins. Data shown were collected up to August 29, 2012, and start dates for serial transfer were as follows: (our *bd*) February 12, 2008, (WT) May 13, 2008, (FGSC *bd*) July 01, 2008, (MC F1) August 18, 2008, (328-4) January 06, 2009, (JD *bd* II) April 8, 2011. Any gaps in the curves represent missing time points. The endpoints are endpoints to data collection. (D) Serial transfer experiments of WT, *lag-1* (NCU00008 F1-1), *bd* (FGSC 1858), and double mutants, *bd*, *lag-1* (-24 or -31^1,2^ in the dark on QA (0.001 mol/L); their average growth rates are 1.81, 1.82, 0.75, 0.42, and 0.46 mm/h. Each time point has 1–12 replicates. WT was the fastest to grow, while the double mutant was the slowest to grow. Light is necessary to trigger conidiation for serial transfer, except for *bd*. After the 5th cycle, the WT culture was exposed to natural light for a few hours to trigger conidiation. The history of growth rates for F1-1 is simply the cumulative amount of data till the time (September 30, 2008) we stopped collecting data. The double mutant *bd, lag-1* did terminate early (i.e., replicatively senesced).

Both the longevity assurance gene (*LAG1)* and the homolog of the mammalian RAS protooncogene (*RAS1)* have been shown to be longevity genes in *S. cerevisiae* (Jazwinski 2002) and to be involved in a transient stress response through sphingolipid metabolites. *LAG1* was the first yeast longevity gene cloned, and its cognate protein was shown to be part of a ceramide synthase (D'Mello et al. 1994). The human homologs, *LASS1* and *HRAS1*, have been established to be longevity genes as well (Jazwinski et al. 2010). In addition, in *N. crassa, ras-1* is the well-known *band* (*bd*) gene, which has been extensively exploited to study the biological clock (Belden et al. 2007). The *bd* gene is probably the oldest example of a *clock-associated gene* in *N. crassa* (Lakin-Thomas et al. 2012). In Figure1A, the results of measuring mRNA levels of *lag-1*^*+*^ mRNA over a 48-hour window are shown. In one microarray experiment, the clock mechanism gene, *white-collar-1* (*wc-1*), was placed under the control of a quinic acid-inducible promoter and switched off at time 0. There was a significant transient response in liquid cultures on the part of *lag-1*^*+*^ mRNA level to the knockdown of *wc-1* (cycle 3 experiment (Dong et al. 2008)). In another microarray experiment, liquid cultures were placed in the dark for 24 h and then shifted to the light for 24 h (cycle 2 experiment (Dong et al. 2008)). Again the mRNA level of *lag-1*^*+*^ displayed a significant transient response.

We ask the following four questions in this study to address the hypothesis in this paper using the strengths of the model system, *N. crassa*: (1) Are there candidate longevity genes under clock control, which are lipid metabolism genes? (2) Do strains of *N. crassa* replicatively senesce as do their relatives *P. anserina* and *N. intermedia*? (3) Do these longevity gene candidates have an effect on chronological aging and other phenotypes? (4) Do the candidate longevity genes interact in their effect on fitness and longevity and with the clock's complex phenotype in particular?

## Materials and Methods^1^

Materials and Methods have the same section headings as the results section to assist the reader in linking results to relevant materials and methods^1^. The gene nomenclatures for *Saccharomyces cerevisiae, Neurospora crassa, and Homo sapiens* are all distinct. For example, the gene *lag-1* in *N. crassa* is uncapitalized and italicized with the cognate protein, all capitals and unitalicized (LAG-1), while in *S. cerevisiae*, the homolog *LAG1* is all capitals, italicized, and has no hyphen, and the cognate protein, first letter capitalized and the rest lower case with no italics (Lag1). The *H. sapiens* homologous gene *LASS1* is italicized capitals and at least 4 letters, while the encoded protein is unitalicized and all capitals (LASS1)

### Replicative life span^2^

#### *N. crassa* strains and genetic crosses

The following strains were used in these experiments: 74-OR23-1A (Fungal Genetics Stock Center (FGSC) 987) known as wild type (WT), *band* (*bd A)* FGSC1858, Dunlap's *band* (*bd A)* denoted 328-4 kindly provided by Jay C. Dunlap, *lag-1*^*KO*^ *a* (NCU00008 and FGSC 13263, knockout derived from *mus-51::bar his-3, A* (FGSC 9718)), and *lac-1*^*KO*^ *a* (NCU02468 and FGSC 13903), and a *clock-controlled gene-2* promoter with luciferase recorder (*bd*,*ccg-2p:luc*) (Gooch et al. 2008). Three different strains of *bd A* were used, an early one from the stock collection (FGSC 1858—which we refer to as our *bd*), a later one from the stock collection (FGSC 1858), and the last strain (328-4) from Jay Dunlap (JD).) Knockout strain *lag-1*^*KO*^ *a* was obtained as a hygromycin-resistant heterokaryon, which was selected as a “knockout” (Colot et al. 2006). This knockout was subjected to two rounds of conidial platings on sorbose, fructose, and glucose (SFG) + fries medium (Davis and de Serres 1970) with selection for hygromycin resistance. The resulting knockout was designated *lag-1-2-2-1*. This *lag-1-2-2-1* strain had very low fertility in crosses on Westergaard (Davis and de Serres 1970). The *lag-1*^*KO*^ (1-2-2-1) was successfully crossed on cornmeal agar (Davis and de Serres 1970) to *his-3, A* – *261* (FGSC 462), a histidine-requiring strain to obtain a homokaryotic isolate and a wild-type hygromycin-resistant strain, NCU00008-F1-1. A replating of this cross-yielded NCU00008-61 F1 *lag-1*^*KO*^*, hy*^*r*^*-resistant his-3* used in transformation experiments in *N. crassa*. The strain NCU00008-F1-1 was crossed to *bd, A* (FGSC 1858) on Westergaard (Davis and de Serres 1970) to obtain *bd, lag-1* hygromycin-resistant strains, -31 and -24.

#### Strains and media for *P. anserina* and *N. intermedia*

*N. intermedia* strains (FGSC 4015 and 3720) were described previously (Bertrand et al. 1986) and were grown on the same media as *N. crassa* strains here. *P. anserina* wild-type strains +F89071 and -F89071 (+ and – indicating mating type) and recommended growth media were provided by courtesy of Dr. Heinz D. Osiewacz. These strains were grown on complete M2 media as defined and modified after Esser (1974). Recommended media consisted of 0.25 g KH_2_PO_4_, 0.3 g K_2_HPO_4_, 0.25 g MgSO_4_x7H_2_0, 0.5 g urea, 10 g dextrin, 20 g agar per liter. To this biotin stock solution (0.05 mg/mL), thiamine stock solution (250 mg/L), and M2/PASM trace element stock solution (10,000 X) were added, which were filter-sterilized. M2/PASM Trace Element stock solution consisted of 5 g citric acid X 1 H_2_0, 5 g ZnSO_4_ × 7 H_2_O, 1 g FE(NH_4_)_2_(SO_4_)_2_ x 6H_2_0, 0.25 g CuSO_4_ × 5 H_2_0, 0.05 g MnSO_4_ × 1 H_2_O, 0.05 g Na_2_MoO_4_ × 2 H_2_O, and 0.05 g H_3_BO_4_ per 100 mL, which was filter-sterilized.

#### Race tube experiments

Race tubes were prepared as described previously (Dharmananda 1980) with modifications listed below. Unless otherwise stated, media used were either Vogel's + 1.5% agar + 0.001M QA + 0.5% arginine or Vogel's + 1.5% agar + 0.15% glucose + 0.5% arginine with amino acid supplements as needed. Conidia were recovered by filtering through glass wool. The choice of QA as a carbon source was driven by more closely approximating growth conditions in nature. Then, 20–25 *μ*L of the filtrant (typically 10^7^ cells/mL) was used to inoculate race tubes containing 20 mL of media. The number of cells in the inoculum has been reported elsewhere to affect growth rate (Richard et al. 2012). Tubes were incubated in the dark at 25°C for at least 4 days and growth fronts, marked each morning usually at 7:30 am under red light. Once cultures reached the end of a tube, they were digitally photographed. Period and phase of race tubes were determined with a modification of a FORTRAN-IV program (Dharmananda 1980) (see Dong et al. (2008)).

#### Serial transfers between race tubes

**Figure 2:**
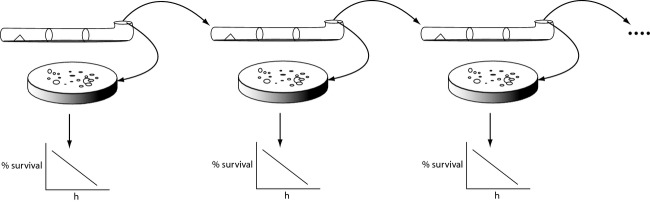
Serial transfer experiment of cultures between race tubes to measure the biological clock, replicative life span, and chronological life span simultaneously.

Once a culture reached the end of a “race tube,” 1 mL of water was used to wash the end of the tube, and 25 *μ*L of the wash with ca 4 × 10^4^ cells was used to inoculate a fresh tube containing 20 mL of media. The old tube was usually allowed to sit on the bench for at least 1 day before taking digital photographs and to allow conidiation before obtaining an inoculum. The light exposure to initiate conidiation was not needed for the *bd* mutant. This serial transfer was continued as in Figure2. Because some of the strains no longer conidiated, a small mycelial plug was taken from the end of the tube (usually from a position under the black cap in Fig.1B.1) and transferred to the next tube (as indicated in Fig.1C by a small white triangle). The use of the mycelial plug in transfer did become the preferred method. All strains were serially transferred in quadruplicate.

### Chronological life span^3^

#### Plating of cell cultures to determine chronological life span

The protocol now described is classic and is distinct from that of Munkres and Furtek (1984a). Cells at cycle 0 in Figure2 were counted using a hemocytometer to achieve a target of 10^7^ cells/mL. These cells were maintained *in water* for 42 days at 25°C to starve them and to measure survivorship. The viability of these cells was determined by plating daily on appropriate medium at a 10^−4^ and/or 10^−5^ dilution as described in Munkres and Furtek (1984a). Colonies were counted after 2- to 3-day growth at 30°C.

### Are there differences in chronological life span between microconidia and macroconidia?^5^

#### Automated cell counting to determine chronological life span

Cells at cycle 0 in Figure2 were counted using a cell counter called a Cellometer Auto 2000 (Nexcelom, Inc Lawrence, MASS USA.) to achieve an initial target of 10^7^ cells/mL for the culture (Berkes et al. 2012). These cells were maintained *in water* for up to 24 days at 25°C in the dark to starve them and to measure survivorship. The viability of these cells was determined by sampling the culture according to a prescribed schedule; samples were stained with 10 *μ*L of 50 *μ*g/mL propidium iodide for 20 *μ*L of cells for 12 min, and then total cell counts and dead cell counts were obtained with an automated cell counter (Nexcelom, Inc.). The settings on the cell counter for bright field were as follows: fluorescent exposure 18,000 msec and dilution factor 1.5. Settings for a bright field view are as follows: roundness 0.1; contrast enhancement 0.7; decluster edge factor 0.5; decluster Th factor 1.0; background adjustment 0.1; sensitivity 6; uniformity 250; and contrast enhancement for dead cells 0.65. Settings for fluorescence are as follows: roundness 0.10; manual threshold fluorescence 8.0; and decluster Th factor 0.5. The cell counter allows control of counting by cell size. For macroconidia, a cell size (alive or dead) of 4.5*–*20 *μ*m was used. For macroconidia+microconidia, a cell size (alive or dead) of 2.2*–*20 *μ*m was used.

### What is the biochemical function of lag-1?^6^

#### Cloning of *N. crassa* lag-1^+^ (NCU00008) and transformation into his-3, lag-1^KO^

The *lag-1* ^*+*^ -coding region was amplified from genomic DNA of OR74A. The PCR product was cloned into pDE3dBH-qa-2 (Cheng et al. 2001) and confirmed by sequencing. The *lag-1*^*KO*^ (*lag-1*-1-2-1) (Colot et al. 2006) was crossed to *his-3, A* (FGSC 261, see crosses above) on cornmeal agar to obtain a homokaryotic *lag-1*^*KO*^ *hygromycin-resistant his-3* (NCU00008-61) strain. This strain was transformed by the spheroplast method with pDE3dBH-qa-2:*lag-1*^*+*^*(Case* et al. 1979). Two of four transformants, T1 (NCU00008-61-T1-1) and T2 (NCU00008-61-T2-2) were made homokaryotic by crossing with *his-3, A* (FGSC 111-6a).

#### *S. cerevisiae* complementation

**Figure 3:**
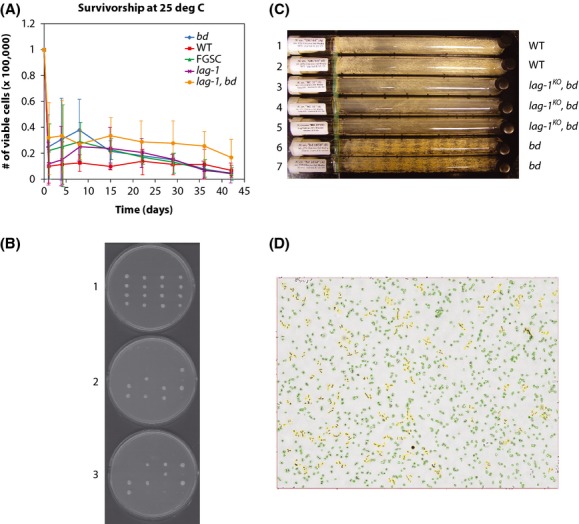
(A) The *bd and lag-1* mutations promote chronological life span in *N. crassa* in three replicate experiments. Two different strains of *bd* were assayed for chronological life span. Our *bd* strain is from the FGSC at an earlier date than the later strain labeled FGSC. These curves represent the averages over three independent replicates of the plating experiment. The error bars were computed from 95% confidence bands under a normal approximation (Draper and Smith 1981) (B) Tetrad dissection of *N. crassa lag-1* transformants in *S. cerevisiae*. Tetrads are arranged vertically (1–4 from left to right). (1) Dissected spores from tetrads grown on a −ura plate. (2) Colonies replicated from A grown on a −ura and −leu plate. (3) Colonies replicated from A grown on a −ura and −trp plate. Carbon sources for media are 2% galactose and 2% raffinose. (C) The *lag-1*^*KO*^*, bd -31* double mutant can stop the biological clock output as displayed in the asexual reproduction of spores along race tubes (3–5) during 7 days of growth from one end to the other end of the race tubes. As a control, a *bd* mutant (FGSC 1858) is shown banding (tubes 6–7), that is, reproducing on a 22-h cycle, while WT (OR74A) is shown not banding (tubes 1–2) under these growth conditions. All tubes were maintained in the dark at 25°C. All strains were grown on glucose (0.15%) as described in the methods. Average growth rates of *bd*, *lag-1*^*KO*^*, bd -31*, and WT were 0.86, 0.99, and 2.20 mm/h on glucose (0.15%). (D) The *bd, lag-1* (−31) double mutant has a chain phenotype with 100× objective. Many of the chains of conidia are colored yellow. Apparently some of the conidia fail to divide successfully.

Functional complementation test of *N. crassa lag-1* (NCU00008) to *S. cerevisiae LAG1*, *LAC1* genes is based on a procedure described previously (Jiang et al. 1998). A ClaI/EcoRI fragment from plasmid pDE3dBH-qa-2: *lag-1*^*+*^(*Jiang* et al. 1998), which contained the *N. crassa lag-1* ^*+*^ -coding region, was cloned into *ClaI/EcoRI* sites of plasmid pRS416 and named pJJ62. Then, a *Kpn1/EcoRI* fragment (*KpnI* just a few bases upstream of the *ClaI* site) of pJJ62 was cloned into *Kpn1/EcoRI* sites of the pBevy-Gu expression vector, which contained the URA3 marker and *GAL1* promoter, and was named pJJ63, putting *lag-1*^+^ behind the galactose-inducible *GAL1* promoter. This newly constructed expression vector was employed to transform an *S. cerevisiae* diploid strain which was heterozygous for a *LAG1* deletion (::*TRP1*) and *LAC1* deletion (::*LEU2*), and its haploid strain with *LAG1*, *LAC1* double deletions was inviable. Transformants were selected on -ura plates. Sporulation of the obtained transformants was carried out on 1% KAC, 0.05% glucose, and 0.1% yeast extract medium, and after four days, tetrads were formed. Tetrads were dissected on -ura plates with 2% galactose and 2% raffinose. These haploid spores were allowed to form colonies (Fig.3B.1). The colonies were replica-plated on -ura, -trp and -ura, -leu plates (Fig.3B.2 and B.3). Colonies grown on both plates had yeast *LAG1* (::TRP1) and *LAC1* (::*LEU2*) double deletions, and their function can be complemented by *N. crassa lag-1* in the expression vector pJJ63 on galactose. Deletions of yeast *LAG1* and *LAC1* and the presence of *N. crassa lag-1* in haploid colonies were also verified by PCR.

## Does lag-1 interact with bd (ras-1) in determining a clock phenotype?^7^

### Real-time quantitative PCR (RT-qPCR)

Cells were harvested at thirteen time points in the dark (D/D) with the design of cycle 1 (Dong et al. 2008). The design enforces a constant growth time of 50 hours on each replicate culture to reach the end at the desired time point (0, 4, 8, …, 48 h). All cells were synchronized by a minimum of 2 h of 70 micromoles per Liter per second per meter squared (*μ*mol/L/sec/m^2^) light exposure before being transferred to the dark. The average time of synchronization in the light across the 13 replicates was 26 h before each replicate was transferred to the dark. RNAs were harvested with a Spectrum Plant Total RNA kit 50 (Sigma Aldrich, St. Louis, MO, USA, Inc.). The integrity, quality, and amount of the total RNAs were assessed with a 2100 Bioanalyzer (Agilent Techologies, Inc., Santa Clara, CA, USA) using the Agilent Technologies, Inc. RNA 6000 Nano LabChip (#5067-1511). From ∼1.5 *μ*g of total RNA, first-strand cDNA synthesis was carried out with a SuperScript III 1st Strand cDNA Synthesis Kit (Invitrogen Inc., Grand Island, NY USA 18080-051) as recommended (Sieber et al. 2010) (Okello et al. 2010). The total RNAs ranged from 0.3 to 1.42 *μ*g because Dr. Dong used a High Pure RNA isolation kit (Roche, Inc. Indianapolis, IN USA) (Dong et al. 2008).

RT-qPCR was carried out in triplicate on each cDNA with *frq* target primers designed by ABI-Prism 7500 software with Brilliant III Ultra-Fast SYBR Green qPCR Master Mix (#600882, Agilent Technologies, Inc.) as recommended (Sieber et al. 2010) (Okello et al. 2010). The endogenous control in triplicate was 18S rDNA (Dong et al. 2008). The use of the 18S rDNA as a reference dates back at least to 1994 (Aronson et al. 1994). The efficiency measures for both primers were correlations of 0.945 (*frq* primer) and 0.957 (rDNA primer) from a calibration curve involving five-, fourfold dilutions (Bustin et al. 2009). Triplicate reactions (25 *μ*L) were analyzed with the ΔΔC_T_ method as implemented on the ABI-Prism 7500 with outlier detection. Six outliers were removed from the 284 wells processed by the ABI software. The relative quantity (RQ) of *frq* mRNA was measured relative to the endogenous control and the zero time point.

## Analysis of variance of log viabilities^4^

The model used to compute the analysis of variance in Table1 is the linear model


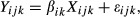
(1)

where *Y*_*ijk*_ is the log viability for the ith strain at time *X*_*ijk*_ for conidia of size *k*. Here, *j* indexes the days. The independent variable *X*_*ijk*_ is the day j for strain *i* of size *k*. The measurement errors *ε*_*ijk*_ are normally distributed with mean 0 and variance *σ*^2^. The parameters *β*_ik_ are the mortality rates on a log-scale, and their estimates are denoted by *b*_*ik*_. Under Hypothesis H_1_, there are no strain differences with *β*_*ik*_ = *β*. Under Hypothesis H_2_, there are strain differences in mortality rates but no differences in mortality rates between micro- and macroconidia with *β*_*ik*_ = *β*_i_. Under Hypothesis H_3_, there are strain and micro-/macroconidia differences in mortality rates with *β*_*ik*_ unconstrained. Models were fitted by least squares and analyzed as described previously (Draper and Smith 1981).

**Table 1:** Analysis of variance of chronological longevity through log viability as a function of age (in days), conidial size, and strain. The dependent variable is log viability as measured from an automated cell counter (Nexcelom, Inc.). Strains (*bd*, WT, FGSC *bd*, *lag-1*, *lag-1, bd,* and *bd,ccg-2p:luc*, (Gooch et al. 2008)) were compared over 9 days.

Source	SS	df	E.M.S.	*F*	*P*
Age	2.0761	1	2.0761	106.47	< 0.01
Between strains	0.3377	5	0.0675	3.46	< 0.01
Between strains and conidial size	0.2056	6	0.0343	1.76	> 0.05
Error	2.4567	126	0.0195		
Total	5.0760	138			

The sums of squares (SS), degrees of freedom (df), estimated mean square (E.M.S.), *F*-ratio (*F*), and *P*-value (*P*) are reported. Their calculation is described (Draper and Smith 1981).

The model in (1) takes the form under a particular hypothesis H_i_ with parameter vector *β*_i_:


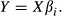
(2)

The parameter estimates *b*_*i*_ were found from the normal equations:


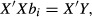
(3)

by inverting *X′X*. The regression sum of squares (*RSS*_*i*_) for each hypothesis *H*_*i*_ was calculated by:


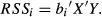
(4)

A telescoping sum of these regression sums of squares was used to form the ANOVA in Table1 to test the difference of one model nested within another model, such as H_1_ versus H_2_. Models were fitted by least squares and nested within each other (H_1_ within H_2_ within H_3_) and hence the significances of differences between H_1_ versus H_2_ and H_2_ versus H_3_ were assessed with an ANOVA (Draper and Smith 1981). This approach is summarized (Draper and Smith 1981), was implemented in FORTRAN-77, and is available on request.

## Analysis of variance of expression of frq mRNA profiles^8^

**Figure 4:**
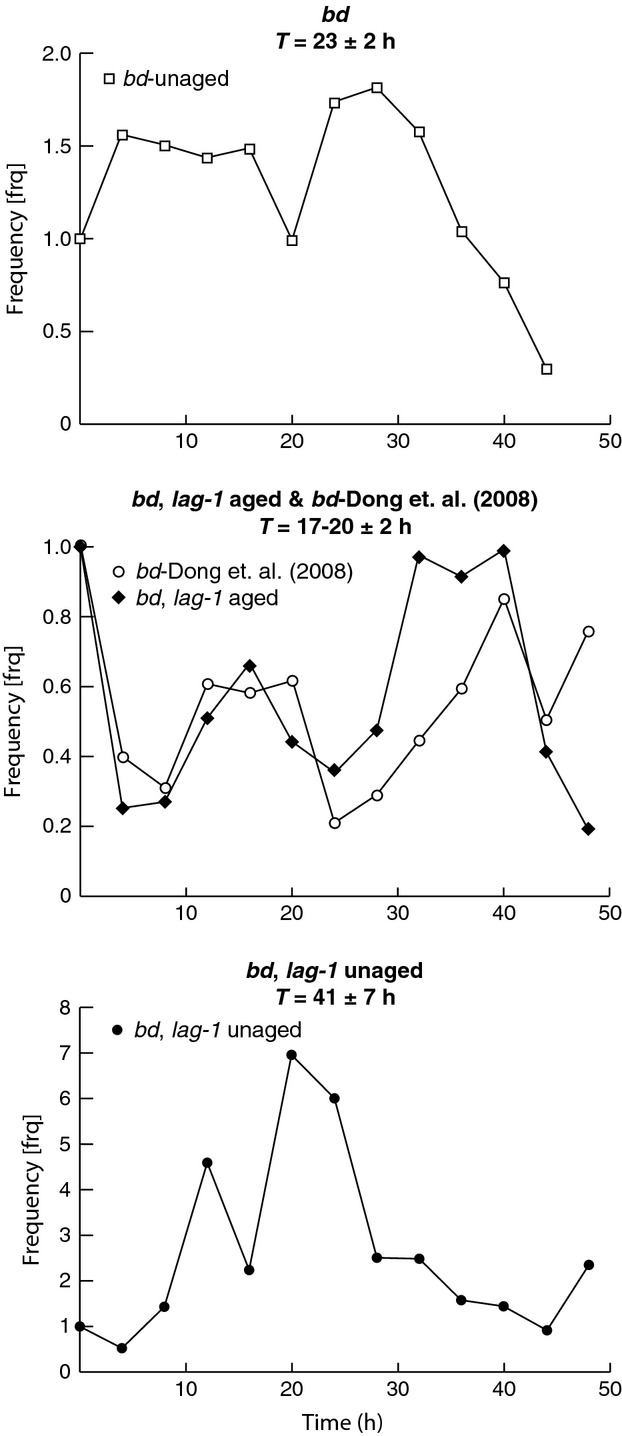
The *bd, lag-1* double mutant affects the clock oscillator (*frq*) expression differentially in aged and unaged cultures of *N. crassa* in the dark (D/D). Two unaged cultures of *bd* are presented as controls with one mRNA profile derived from earlier work (Dong et al. 2008). The estimated periods (T_i_) for each culture are reported as an inset with standard error. The *y*-axis is the relative quantity (RQ) of *frq* mRNA as measured by RT-PCR using 18S rRNA as an endogenous control, and the *x*-axis is time in hours (h).

The nonlinear model used to compute the analysis of variance in Table3 is


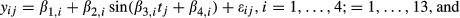
(5)

time points *t*_*j*_ = 0, 4, 8,…,48 h. The dependent variable *y*_*ij*_ is the relative expression (RQ) of the *frq* mRNA in the *i*th strain at the *j*th time point. There are 4 strains in Figure4, and there are 13 equally spaced time points *t*_*j*_ = 0, 4, 8, …, 48 h. The errors (*ε*_ij_) are assumed to be independently and identically distributed *N*(0, *σ*^2^) random variables. In this model, each strain is characterized by a fixed y-intercept (*β*_1,*i*_), amplitude (*β*_2,*i*_), frequency (*β*_3,*i*_ with the inverse proportional to period), and phase (*β*_4,*i*_).

Under Hypothesis 1 (single oscillator), the 4 strains have the same oscillator and *β*_k,I_ = *β*_κ_ for all *k* = 1,…,4. Under Hypothesis 2 (two oscillators), 3 strains are characterized by the same oscillator, and *β*_k,1_ = *β*_κ,2_ = *β*_κ,3_. Under Hypothesis 3 (3 oscillators), 2 strains are characterized by the same oscillator, and *β*_k,2_ = *β*_κ,3_, *k* = 1,…4. Under Hypothesis 4 (four oscillators), 4 strains are characterized each by a distinct oscillator, and the *β*_*k,i*_ are unconstrained.

Each of the four models was fitted by the method of maximum likelihood. The maximum-likelihood estimates were computed by maximum-likelihood scoring. The score is defined to be *S*_*k*_ = ∂ln *L*/ ∂*β*_*k*_, and the information matrix, to be *I*_*kl*_ = – *E*(∂^2^ ln *L*/ ∂*β*_*k*_ ∂*β*_*l*_). An update (a *p* × 1 dimensional vector) *δ* was solved from


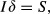


where *I* is the *p* × *p* information matrix and *S* is a *p* × 1 vector of scores for each of *p* parameters. The parameter vector *β* at the next iteration was updated to *β*' = *β* + *δ*. The updating continued until a tolerance of 10^−5^ was achieved. This happened in less than 50 iterations. The asymptotic variance–covariance matrix of the parameter estimates in (5) was calculated from *I*^−1^.

Once the parameters were estimated, the parameters of frequency (*β*_3,*i*_) and phase (*β*_4,*i*_) were treated as known. The quantity sin(*β*_3,*i*_*t*_*j*_ +*β*_4,*i*_) in (5) was treated as an independent variable in a linear regression. The resulting linear regression in the amplitude(s) and y-intercept (s) was performed, and a regression sum of squares (RSS) was computed from (4). Each of the four models then generated an RSS and associated error sum of squares (ESS) to produce the telescoping sums of squares in Table3. Maximum-likelihood scoring was implemented in Fortran-77. As a control to check the scoring procedure, a separate set of Fortran-77 programs was written to perform a grid search for the period and phase followed by a linear regression using the normal equations in (3) to obtain the *y*-intercept(s) and amplitude(s).

## Results

All results are cross-indexed to Materials and Methods^1.^

The first prediction of the hypothesis in this paper is that some lipid metabolism genes in *N. crassa* should be longevity genes. Aging can be measured in two ways by replicative life span and chronological life span. Replicative life span is defined as the number of serial transfers completed before the strain dies, as shown in Figure2. Chronological life span is defined to be how long a conidium lives for a particular strain in a particular medium. A conidium could enter a state of low metabolic activity and persist in the environment for many days. In contrast, in a race tube, a conidium needs to be able to grow, develop hyphae, and replicate nuclei to reach the other end of the race tube for serial transfer. These two measures of successful aging assess different activities of the organism and involve different suites of genes to carry out these activities in *S. cerevisiae* (Stumpferl et al. 2012).

### Replicative life span^2^

Aging was measured in *N. intermedia* and *Podospora anserina* (relatives of *N. crassa*) by determining the replicative life span in a series of “race tube” experiments (Fig.2). These experiments are shown in Figure1B.1 where *P. anserina* did not even complete one cycle (growth to the end of the race tube) with the strains examined. For *N. intermedia,* it took 4 cycles before the inserted aging-inducing plasmid (Fig.1B.2) in the mitochondria in the Kalilo strain 5014 led to senescence (Griffiths 1992) while the WT strain 3720 without the plasmid continued to grow. The question is whether or not *N. crassa* experiences replicative senescence. Initially, there did not appear to be a replicative life span for seven strains in Figure1C after 100 cycles of serial transfer. The only strain examined here that appeared to senesce after 3 serial transfers was the double mutant *lag-1, bd* (yellow and green curves in Fig.1D) as in strains with the Kalilo plasmid (Bok et al. 2003).

### Chronological life span^3^

To measure chronological life span, survivorship curves were determined for WT, *lag-1*^*KO*^*-1*, *bd*, and a double mutant *lag-1*^*KO*^*, bd* -31^1^ by a serial dilution assay to 10^−5^, and plating conidia to count survivors on sorbose, fructose, glucose media (SFG) transferred from starvation media (water at 25°C) over a 42-day period^3^. Both *ras-1* (*bd*) and *lag-1* acted as chronological longevity genes in three independent replicate experiments in Figure3A, with lessened decay in survivorship over time relative to WT. This is as expected from results in *S. cerevisiae* (D'Mello et al. 1994). Thus, both *bd* (*ras-1*) and *lag-1* affected chronological life span in *N. crassa*.

Variation in measuring viability and longevity is a general problem (Anderson et al. 1986). The longevity schedules in Figure3A have considerable variation by the traditional plating method due to heterogeneity in the population measured, the growth media selected, and/or measurement error through the plating of a serial dilution of conidia. There are at least two kinds of conidia, microconidia (2.5–3.5 *μ*m) and macroconidia (5–9 *μ*m) (Maheshwari 1999). As an example of a growth media effect, survival curves for microconidia were observed by plating fluffy mutants (*fl*) and differed between growth media (Barratt 1964).

Methods for measuring conidial chronological life span have remained unchanged for 50+ years in *N. crassa*, the time that the senior author has worked on *N. crassa*. New methods for measuring aging are needed in fungi. Here, we used a Cellometer Auto 2000 (Nexcelom, Inc.) or automated cell counter to measure longevity of conidia sorted by size to test the hypothesis that size explains the noise in Figure3A.^5^

In Table1, there was a significant difference in chronological life span between the five strains, shown in Figure3A, as measured using a fresh approach with an automated Cellometer.^5^ A similar result was found for the classic plating method. In order to assess the variability in the plating vs. the automated cell-counting method, we added two more plating replicates to those used in Figure3A in Table2. For example, the WT macroconidial estimated median life span of 24 days measured by the automated Cellometer was consistent with prior measurements (Munkres and Furtek 1984c), but the estimated macroconidial median life span of the double mutant *lag-1, bd* was 120 days in Table2. In general, the pattern of variation across strains was consistent whether measured by automated cell counting of macroconidia or plating in Table2. The major difference was that 5 replicates of a plating experiment in Table2 yielded similar standard errors on the mortality rates as one replicate by automated cell counting of macroconidia. We conclude that controlling conidial size by limiting longevity measurements to macroconidia reduces the variation in estimates of longevity (Table2).

**Table 2:** Estimates of mortality rates (with standard errors given in parentheses) per day (log-scale for viability), expected life span, and expected median life span for varied strains (our *bd*, wild type (WT), FGSC *bd*, *lag-1*, *lag-1,bd*, and *bd,ccg-2p:luc* (Gooch et al. 2008)) over 9 days. The R^2^ is the fraction of variation in an ANOVA (see Table1) explained in a regression of log viability on day, conidial size, and strain. The number of time points or sample size is *n*.

Method	*R*^2^	Our *bd*	WT	FGSC *bd*	*lag-1*	*lag-1, bd*	*bd,ccg-2p: luc*	*n*
Plating	0.71							300
Mortality rate per day b		−0.0785 (0.0080)	−0.0844 (0.0076)	−0.0896 (0.0074)	−0.0854 (0.0075)	−0.0604 (0.0071)	–	
Expected life span (-1/b)		13 days	12	11	12	17	–	
Median life span(-ln 2)/b		9 days	8	8	8	12	–	
Microconidia + macroconidia from automated cellometer	0.49							
Mortality rate per day b		−0.0306 (0.0095)	−0.0284 (0.0095)	−0.0256 (0.0108)	−0.0634 (0.0095)	−0.0181 (0.0108)	−0.0492 (0.0108)	69
Expected life span (-1/b)		33	35	39	16	55	20	
Median life span(-ln 2)/b		23	24	27	11	38	14	
Macroconidia alone from automated cellometer	0.60							
Mortality rate per day b		−0.0272 (0.0075)	−0.0283 (0.0075)	−0.0150 (0.0063)	−0.0279 (0.0075)	−0.0058 (0.0063)	−0.0433 (0.0063)	69
Expected life span (–1/b)		37	35	67	36	172	23	
Median life span (–ln 2)/b		25	24	46	25	120	16	

### Are there differences in chronological life span between microconidia and macroconidia?^5^

Here, we used a Cellometer Auto 2000 (Nexcelom, Inc.) or automated cell counter to measure chronological life span of conidia sorted by size to test the hypothesis that size is an explanation for the noise in Figure 3A^5^. In Table1, testing revealed no differences in mortality rates due to conidial size as measured using the automated Cellometer from an analysis of variance (ANOVA)^4^. The model underlying the analysis of variance in Table1 was the linear model.^4^

Three models were tested in an analysis of variance in Table1.^4^ There were significant strain differences in mortality rates, but no significant difference in mortality rates due to conidial size. When both microconidia and macroconidia were counted, there was more variation in the data as measured by the lower R^2^ in Table2. We conclude that controlling conidial size by limiting longevity measurements to macroconidia reduces the variation in estimates of longevity (Table2). In fact, there was a significant cross-replicate component of the ANOVA in plating experiments. The linear model fitted the log-viabilities quite well (R^2^ in the range 0.6–0.7, independent of method for measuring viability). Lastly, the pattern of mortality in microconidia + macroconidia still revealed the interaction between *lag-1* and *bd* with respect to longevity, and with the exception of WT, the mortality rates were higher than for macroconidia (Table2).

### What is the biochemical function of lag-1?^6^

To test the biochemical function of the *N. crassa lag-1 (*NCU00008), the *lag-1*^+^ gene was cloned into plasmid pDE3dBH-qa-2 (Cheng et al. 2001) and transferred into a yeast expression vector for transformation of *S. cerevisiae*. There was another homolog of *LAG1* in *S. cerevisiae*, called the longevity assurance gene cognate (*LAC1)*. The proteins Lag1 and Lac1 have been shown to function as part of a ceramide synthase complex in *S. cerevisiae* (Guillas et al. 2001) (Schorling et al. 2001). Only with a double mutant was a phenotype seen in *S. cerevisiae* (Guillas et al. 2001). We now establish that *lag-1* in *N. crassa* functionally complements both *LAG1* and *LAC1* in *S. cerevisiae*.

In Figure3B, we observed the successful transformation of *S. cerevisiae* with the *N. crassa lag-1*^*+*^ allele (URA3 marker). In the replicate plates (Fig.3B.2 and B.3), we also observed that spores (column 1 and column 4) that possess *TRP1*, *LEU2,* and *URA3* markers did form colonies. The cells in these colonies expressed *N. crassa lag-1*^*+*^ from the *URA3*-containing vector, and they had yeast *LAG1* and *LAC1* double deletions, tagged by *TRP1* and *LEU2,* respectively. As this haploid strain was inviable with *LAG1* and *LAC1* double deletions, it is reasonable to conclude that these colonies' formation is because *N. crassa lag-1*^*+*^ has successfully complemented *LAG1* and *LAC1* function in *S. cerevisiae*. The *N. crassa lag-1* (NCU00008) is then able to restore the function of either *LAG1* or *LAC1*, a first step in establishing the encoded biochemical function of *lag-1*.

### Does lag-1 interact with bd (ras-1) in determining a clock phenotype?^7^

With a possible interaction between *bd* and *lag-1* in determining chronological life span established in Figure3A and Table2, we now test that aging and the clock are linked through lipid metabolism by asking how these two genes interact to affect a major complex trait with a biological clock in *N. crassa*, namely the asexual reproduction (conidiation) on a 22-h cycle. Race tubes were set up for the double mutant, a *lag-1*^*KO*^ *bd -31*^2^. The results are shown in Figure3C. As a control, the double mutant was crossed with WT to check that isolates segregated for *bd*. The *lag-1* KO phenotype was confirmed by growth on hygromycin (200 *μ*g/mL). Relative to the control *bd*, the double mutant displayed no clock phenotype (i.e., no banding of the conidia) in Figure3C. Conidiation (asexual reproduction) no longer had a clock phenotype. In that both *lag-1* and *bd* (*ras-1*) are clock-associated genes and in that *lag-1* and *bd* (*ras-1*) are chronological longevity genes, we have established that these two longevity genes together can have an impact on a clock phenotype.

While the *bd, lag-1* mutant affects the clock phenotype in Figure3C, it may or may not affect the clock mechanism itself. The *frequency* (*frq*) gene is hypothesized to be part of the clock mechanism and the oscillator for the system (Yu et al. 2007). To test which of these two possibilities holds *frq* oscillator expression was examined directly in the *bd, lag-1* double mutant to see whether *frq* expression is perturbed in aged and unaged cultures in the dark (D/D) for up to 48 h, a “cycle 1” experiment (Dong et al. 2008). In such an experiment, cells were grown for a constant period of 50 hours, light-synchronized for an average of 26 h, and were then transferred to the dark (D/D).^7^ An aged culture was maintained in water for 30 days^2^ before initiating the cycle 1 experiment. As a control to the unaged culture, we also observed the single mutant *bd* under the same conditions (D/D). Measurements of *frq* expression in *bd, lag-1* were carried out by RT-PCR over a 48-h window in liquid cultures in the dark^7^ in aged (30 days) and unaged cultures (1). To assess the effects of *bd, lag-1* on the *frq* oscillator, a simple nonlinear model was fitted to each *frq* RNA profile of the four strains in Figure4.^8^ In this model, each strain has a fixed period, amplitude, phase, and y-intercept estimated by the method of maximum likelihood^8^ under four hypotheses about the similarities of the oscillator in each strain. The results are in Figure4 and Table3 and supported the oscillator being different in aged and unaged cultures. We conclude that *bd, lag-1* affects both the clock phenotype and the clock oscillator and that this effect depends on the age of the culture.

**Table 3:** Expression of the clock oscillator *frq* varies with age in the double mutant *bd, lag-1*. The four experiments are indexed by i, namely *frq* mRNA profiling on: (*i* = 1) *bd* unaged culture assayed in tandem with experiments (2) and (4); (*i* = 2) *bd, lag-1* aged culture assayed in tandem with experiments (1) and (4); (*i* = 3) *bd* unaged culture assayed in 2008 and reported in Dong et al. (2008); (*i* = 4) *bd, lag-1* unaged cultured assayed in tandem with experiments (1) and (2). Four nonlinear models with a specified period, phase, amplitude, and *y*-intercept for each strains were fitted to the RNA profiling data in Figure4 by the method of maximum likelihood: (one oscillator) *β*_k,I_ = *β*_κ_; (two oscillators) *β*_k,1_ = *β*_κ,2_ = *β*_κ,3_; (three oscillators) *β*_k,2_ = *β*_κ,3_; (four oscillators) *β*_k,I_ unconstrained^8^.

Source	df	SS	E.M.S.	*F*	*P*
One oscillator	4	82.0130	20.5033	11.00	< 0.001
Two vs. one oscillator	4	19.5283	4.8821	2.6198	= 0.05091
Three vs. two oscillators	4	1.5961	0.3990	0.21	> 0.05
Four vs three oscillators	4	0.0138	0.0035	.0019	> 0.05
Error sum of squares for 4 separate oscillators	36	67.0852	1.8635		
Total	52	170.2363			

*R*^2^ = 0.61.

The results in Figure4 indicate that there is a substantial difference in oscillator behavior in aged and unaged cultures for the double mutant. The period of oscillation for the unaged culture is estimated at 41 h, while the periods of the remaining three profiles are not significantly different from (at the 0.05 level) the 22-h period from race tube cultures of *bd* (See Fig.3C). A system in which the period of oscillation is nearly as long as the period of observation on the same system cannot be distinguished from a nonoscillatory system. Such a long-period estimate in *frq* is consistent with stopping the clock oscillator *frq* and with the loss of driven oscillations in race tubes (Fig.4C). While the 95% confidence band on the core clock oscillator *frq's* period (namely T in *β*_3,I_ = 2*π*/*Τ*_*i*_) for the unaged double mutant contrasts sharply with the periods of the remaining cultures, an ANOVA was still performed using the above nonlinear model to test the significance of this difference further. The results in Table3 suggest that the best model was one in which the unaged *lag-1,bd* culture had a separate period (y-intercept (*β*_1,i_), phase (*β*_4,i_), and amplitude (*β*_2,i_)), and the remaining cultures were treated as replicates with the same y-intercept, amplitude, phase, and period. We conclude that *bd, lag-1* affects both the clock phenotype and the clock oscillator and that this effect depends on the age of the culture.

## Discussion

There are very few studies on the demography of fungi particularly in natural populations (Pringle and Taylor 2002). Exceptions are longevity studies of *S. cerevisiae* beginning with the longevity assurance gene (*LAG1*) (D'Mello et al. 1994) and more recently on *SIRT1* (Stumpferl et al. 2012), studies of conidial longevity (Munkres and Furtek 1984c) (Munkres and Furtek 1984b), programmed senescence in Neurospora through the Kalilo element (Griffiths 1992) (Bertrand et al. 1986), and senescence in Podospora (Osiewacz 2002). This seems somewhat surprising given the tractability of microbial systems and the emergence of common mechanisms of aging from aging studies of yeast to humans (Fontana et al. 2010). For example, the *lag-1* and *ras-1* (*bd*) genes and their interaction in determining longevity appeared conserved from *S. cerevisiae* (D'Mello et al. 1994) and *N. crassa* (Fig.3A) to *H. sapiens (Jazwinski* et al. 2010). The *lag-1* gene, for example, appeared to encode a ceramide synthase in all three species with the *N. crassa lag-1* complementing both *LAG1* and *LAC1* in *S. cerevisiae* (e.g., Fig.3B).

We have shown that the human homologs, *LASS1* and *HRAS1*, acted in very similar ways to their counterparts, *lag-1* and *ras-1*, in *N. crassa* (Jazwinski et al. 2010). Both *LASS1* and *HRAS1* and *lag-1* and *ras-1* acted epistatically to extend chronological life span (Tables2 and 4). Both sets of genes complemented their yeast homologs, *LAG1* and *RAS1* (Jiang et al. 1998), implying a common biochemical function in sphingolipid metabolism. In humans, we hypothesized that *LASS1* and *HRAS1* respond to lipotoxicity to promote chronological longevity. In *N. crassa,* the double mutant *lag-1*, *bd* (*ras-1*) apparently suffered defects in the lipid rafts of cell walls (London and London 2004), preventing some conidia from proper separation (Fig.3D). These parallels involving the same genes in lipid metabolism would argue for a common mechanism of aging that is highly conserved from fungi to humans (Table4).

**Table 4:** The known effects of yeast *LAG1*, *RAS1*, and *RAS2* and their homologs on life span. The double mutant in *N. crassa* is distinguished as (*lag-1*, *ras-1*). Genes without a superscript were assigned a longevity phenotype here.

Lifespan	*S. cerevisiae*	*N. crassa*	*H. sapiens*
Replicative	*LAG1* ^(D'Mello et al.^ 1994^)^, *RAS1* ^(Jazwinski^ 2002^)^, *RAS2* ^(Jazwinski^ 2002^)^	(*lag-1, ras-1*)	
Chronological	*RAS2* ^(Fabrizio et al.^ 2003^)^	*lag-*1, *ras-1*	*LASS1* ^(Jazwinski et al.^ 2010^)^, *HRAS1* ^(Jazwinski et al.^ 2010^)^

The longevity of *N. crassa* did not simply appear to be an output of the clock through *ras-1* (*bd*) and *lag-1* (Fig.3A). Mutations in the longevity genes, *lag-1* and *ras-1* (*bd*) together, stopped the clock associated with conidiation (Fig.3C) and lengthened the periodicity of the clock oscillator to the point of being indistinguishable from a system lacking a clock (Fig.4). These results are not without precedent. Both *chol-1* and *cel-1* in *N. crassa* glycerophospholipid metabolism and fatty acid biosynthesis also both affected the periodicity and temperature compensation in the clock (Lakin-Thomas and Brody 2000). Here, we have established that two fundamental processes, aging and the clock, are linked by the epistatic interactions between two genes in sphingolipid metabolism. This lends support to the hypothesis that aging and circadian rhythms are mutually linked through lipid metabolism in a common stress response.
